# An Atypical Presentation of Sweet's Syndrome in a Patient With Neutropenia: A Case Report

**DOI:** 10.7759/cureus.79023

**Published:** 2025-02-14

**Authors:** Thanh-Phuong N Afiat, Samuel Jalali, Chandler McCain, Amy Jayson, Hardik Thakkar, Kevin Eaton, Timothy N Hembree, Neha Verma

**Affiliations:** 1 Internal Medicine, Moffitt Cancer Center, Tampa, USA; 2 Internal and Hospital Medicine, Moffitt Cancer Center, Tampa, USA

**Keywords:** acute febrile neutrophilic dermatosis, fever rash, gomm-button disease, neutropenic rash, neutrophilic dermatosis, sweet syndrome

## Abstract

Sweet syndrome (SS), or acute febrile neutrophilic dermatosis, is a non-infectious inflammatory skin reaction characterized by the abrupt onset of tender, erythematous skin lesions commonly appearing on the head, neck, trunk, and upper extremities. The condition remains rare and multifaceted, often posing a diagnostic challenge. There is a broader spectrum of SS than previously recognized, encompassing a variety of overlapping features, clinical and histological variants, and associated diseases. We report an atypical presentation of Sweet Syndrome in a 54-year-old female with neutropenia and myelodysplastic syndrome to enhance awareness and management of this rare condition among hospitalists. It demonstrates how SS can develop in the context of chemotherapy-induced neutropenia, thereby highlighting the importance of recognizing this case presentation in relevant clinical scenarios.

## Introduction

"Acute febrile neutrophilic dermatosis" was first eponymously termed "Sweet Syndrome" in 1964, following a case series by Dr. Robert Douglas Sweet [1]. This series described eight women who presented with fever, neutrophilic polymorphonuclear leukocytosis, painful plaques on the extremities, face, and neck, and histological findings of dense dermal infiltration with mature neutrophil polymorphs [1,2]. It was also formerly referred to as Gomm-Button disease, named after the first two patients diagnosed with Sweet Syndrome (SS) [3].

SS is a rare and complex condition of unknown incidence that can be diagnostically challenging due to its diverse features, associated diseases, and uncertain pathogenesis [2]. Additionally, new therapeutic options beyond traditional corticosteroids are increasingly being explored. In this report, we present a case of SS designed to educate hospitalists on recognizing and managing this unusual disorder. Furthermore, it highlights the possibility of SS presenting in conjunction with chemotherapy-induced neutropenia, thereby increasing awareness of this condition in such clinical contexts.

## Case presentation

A 54-year-old female patient with myelodysplastic syndrome (MDS), and no other past medical history, who started treatment with azacytidine three weeks prior, presented to Urgent Care with a three-day history of bilateral knee pain, intermittent fevers, and right conjunctival erythema for one day. Two days prior, she had been seen in the clinic for the bilateral knee pain and was prescribed loratadine to address the bone pain associated with count recovery. She had received azacitidine with palonosetron pre-treatment only and was not started on any other medications, besides loratadine.

Vitals signs revealed a temperature of 99.9℉ and a heart rate (HR) of 107 beats per minute (bpm). The physical exam was unremarkable with a normal musculoskeletal exam, right conjunctival erythema, and no rashes or dermal lesions. Laboratory data showed a low white blood cell count (WBC) of 2.19 k/uL and a low absolute neutrophil count (ANC) of 1.25 k/uL. Additionally, the erythrocyte sedimentation rate (ESR) was elevated at 83 mm/hour, and the C-reactive protein (CRP) level was elevated at 10.51 mg/dL (Table 1). 

**Table 1:** Laboratory workup prior to and during admission

Laboratory Tests	Patient values four days prior to admission	Patient values Hospital Day 1	Patient values Hospital Day 2	Units	Reference Range
White Blood Cell Count	2.19	1.75	0.84	k/uL	4.0-10.9
Absolute Neutrophil Count	1.25	1.19	0.4	k/uL	1.8-7.8
Relative Neutrophils	57	68	48	%	40.0-80.0
Absolute Eosinophil Count	0	0	0	k/uL	0.0-0.45
Relative Eosinophils	0	0	0	%	0.0-7.0
Absolute Basophil Count	0	0	0	k/uL	0.0-0.2
Relative Basophils	0	0	0	%	0.0-2.0
Absolute Monocyte Count	0.02	0	0.01	k/uL	0.3-0.8
Relative Monocytes	1	0	1	%	0.0-12.0
Absolute Lymphocyte Count	0.92	0.56	0.32	k/uL	1.1-3.5
Relative Lymphocytes	42	32	38	%	15.0-45.0
Absolute Immature Granulocytes	0	0	0.11	k/uL	0.0-0.10
Relative Immature Granulocytes	0	0	13	%	0.0-1.0
Erythrocyte Sedimentation Rate	83	124	91	mm/hr	0-20
High Sensitivity C-Reactive Protein	10.51	17.67	7.42	mg/dL	0.0-0.5

Bilateral knee and chest radiographs were within normal limits. The infectious workup, which included tests for lactic acid, procalcitonin, a viral respiratory panel, and blood cultures yielded negative results. Given the unremarkable findings, the patient was discharged home from Urgent Care with instructions for supportive management.

**Figure 1:**
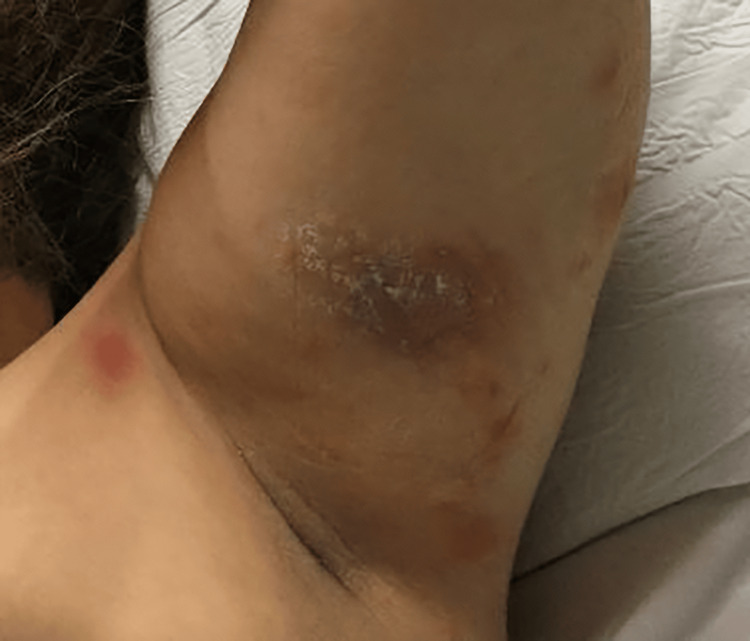
Tender, erythematous, and edematous papulonodules scattered over left axilla.

**Figure 2:**
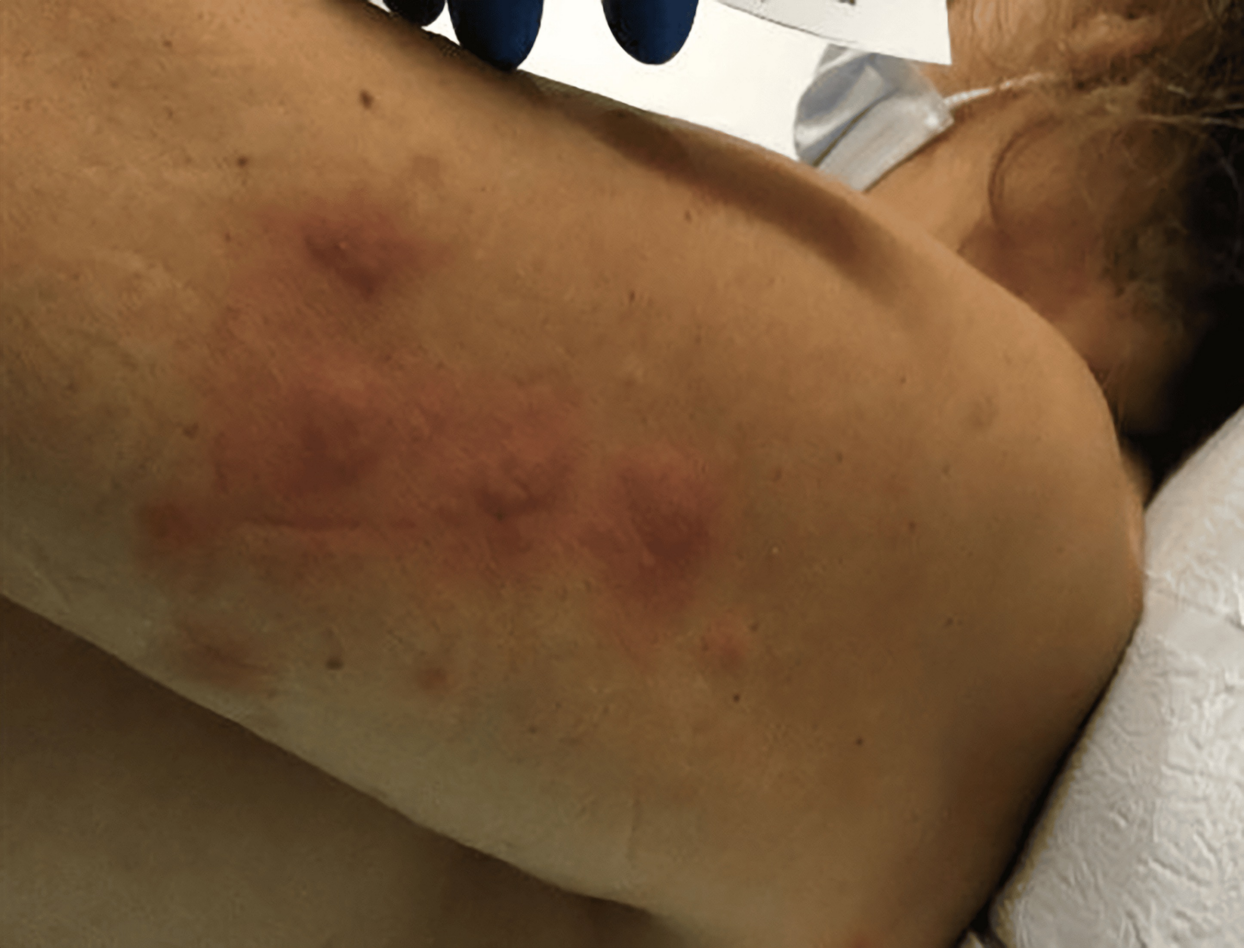
Tender, erythematous, and edematous annular plaques scattered over left lateral arm.

**Figure 3:**
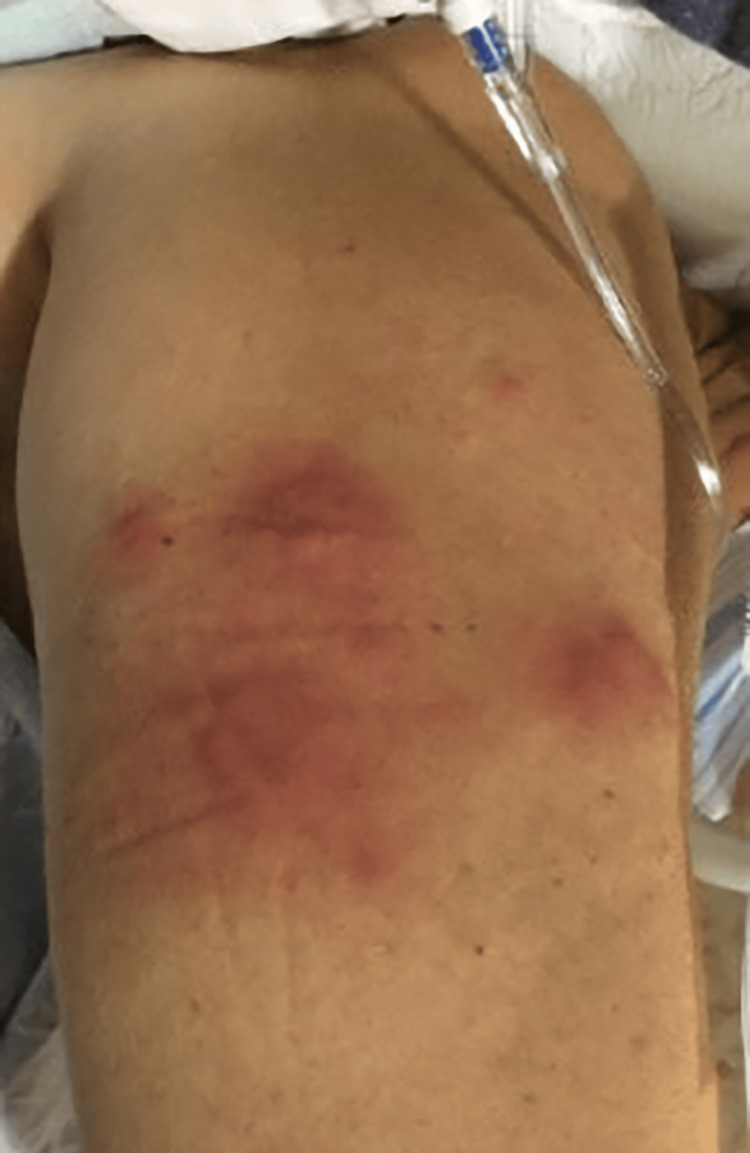
Tender, erythematous, and edematous annular plaques scattered over the right lateral arm.

**Figure 4:**
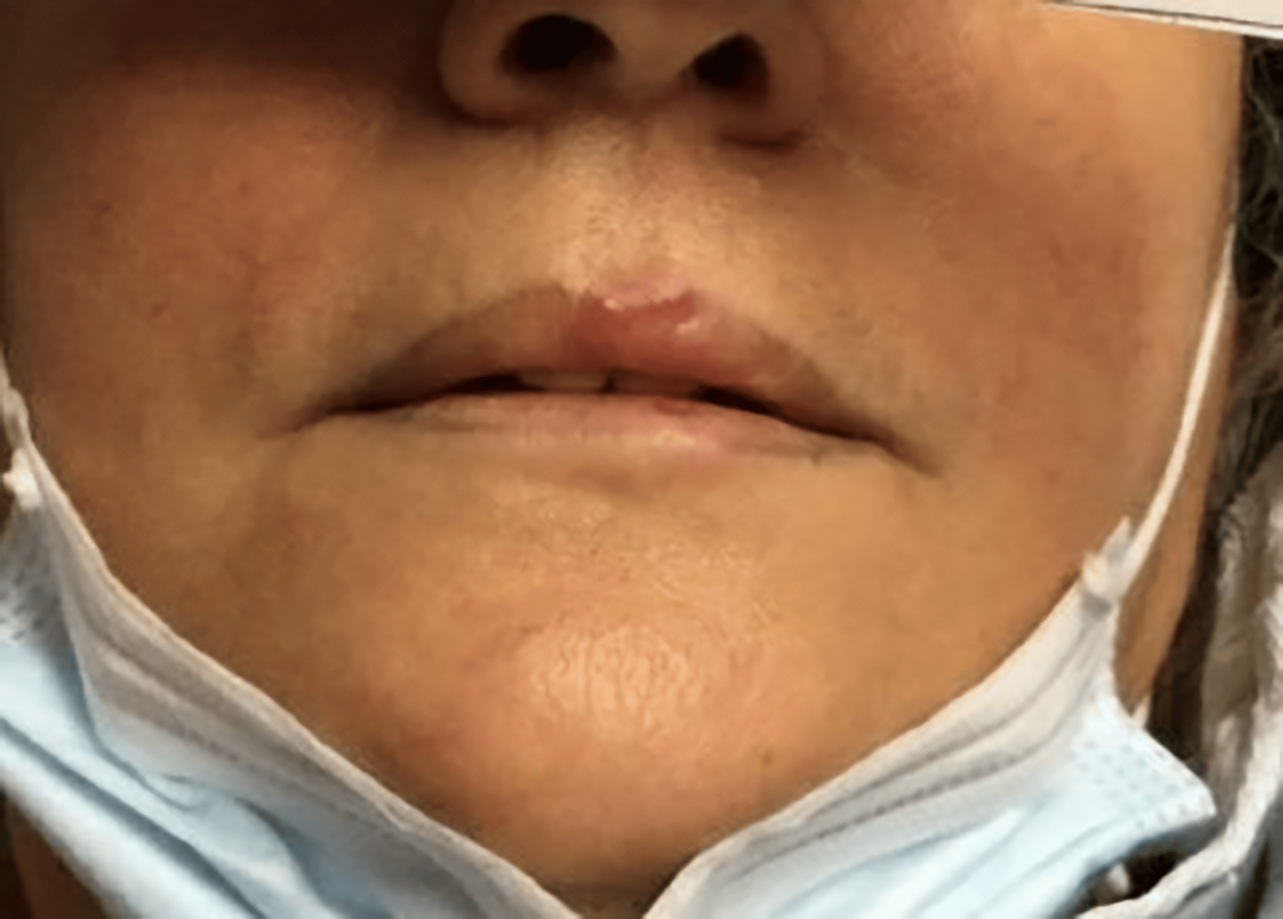
Tender and erythematous papules overlying the upper vermillion border.

**Figure 5:**
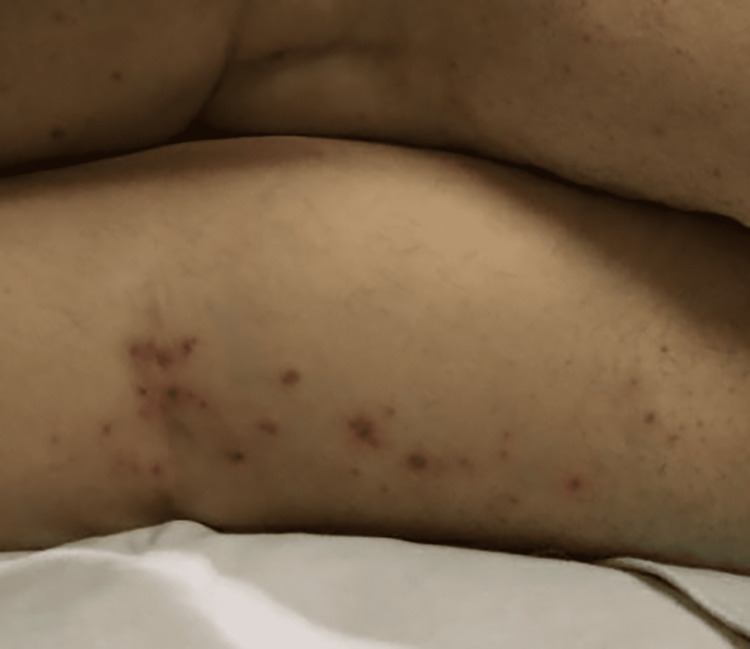
Tender, erythematous papules scattered over the right posterior thigh.

However, four days later, the patient returned to the urgent care with significant worsening of bilateral knee pain affecting mobility, persistent fevers reaching up to 104℉, bilateral conjunctival erythema, and the onset of a new tender rash two days ago. The rash initially appeared on her upper extremities (Figures 1-3) and rapidly disseminated to involve her trunk, face (Figure 4), and lower extremities (Figure 5).

Vital signs at this admission included a temperature of 101º F and a heart rate of 110 bpm. The physical exam revealed tender, erythematous papules, nodules, and plaques on the bilateral upper and lower extremities, as well as the face and scalp. Additionally, the patient had appreciable bilateral conjunctivitis. Laboratory results showed a WBC count of 1.75 k/uL with a low neutrophil count of 1.19 k/uL, an elevated ESR of 124 mm/hour, and a CRP level of 17.67 mg/dL. Subsequent laboratory tests revealed severe leukopenia and severe neutropenia, with a WBC count of 0.84 k/uL and ANC of 0.40 k/uL. 

Due to the patient’s neutropenic fever and severe neutropenia, she was initially prescribed intravenous cefepime, and Infectious Disease was consulted. Despite an extensive infectious workup, including chest radiographs, blood cultures, urinalysis, and a viral respiratory panel, all tests remained negative. Repeat bilateral knee radiographs demonstrated no osseous abnormalities but did reveal a new small suprapatellar knee joint effusion.

**Figure 6:**
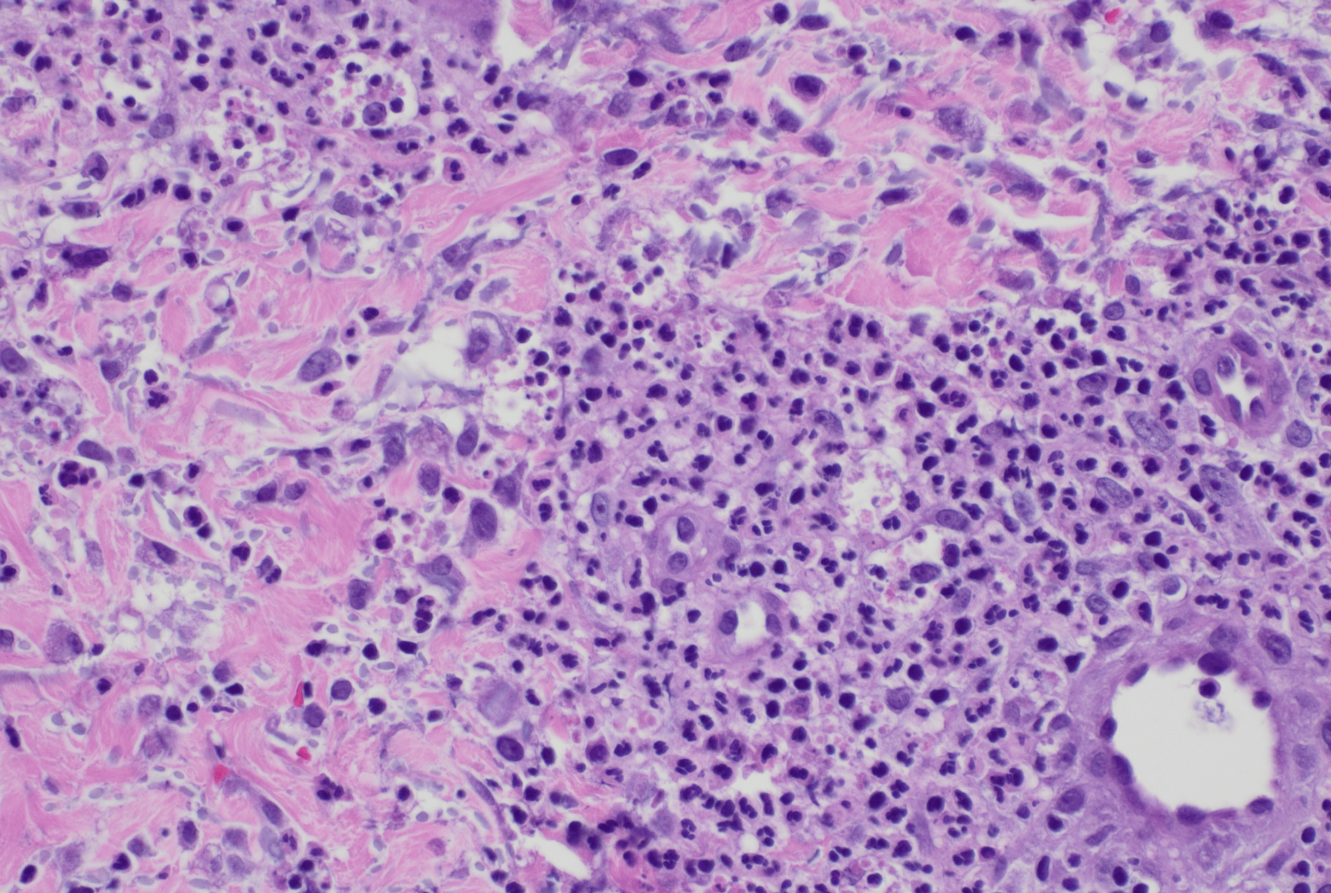
Skin biopsy Skin biopsy revealed peri-vascular, interstitial, and peri-adnexal neutrophilic infiltration of the dermis with rare red blood cells present. The blood vessels within the dermis were dilated with leukocytoclasis present. However, there was no evidence of significant vascular damage or red blood cell extravasation to suggest vasculitis. The epidermis was not involved with slight spongiosis and underlying papillary dermal edema.

At this point, an infectious etiology was deemed less likely. Given the patient’s presentation and objective findings, dermatology was consulted with a suspicion of SS. The patient was admitted and immediately started on prednisone 80 mg daily on the day of admission with a planned taper over four to six weeks. Notably, within 24 hours, there was a marked improvement in her symptoms. She was discharged four days after admission with instructions to complete the steroid taper. The diagnosis of SS was confirmed by the patient’s positive response to steroids and a biopsy of a cutaneous lesion that revealed neutrophilic dermatosis (Figure 6). 

## Discussion

SS is a rare inflammatory disorder classified into three primary subtypes based on etiology: classical, malignancy-associated, and drug-induced. Classical SS occurs in patients without a history of malignancy or drug exposure. Malignancy-associated SS is commonly observed in patients with hematologic malignancies, particularly acute myeloid leukemia (AML), or myeloproliferative disorders, rather than solid tumors. Drug-induced SS typically arises about two weeks after drug exposure, with granulocyte colony-stimulating factor (GCS-F) identified as the most common causative agent. SS remains a rare condition, but its spectrum is expanding to include associations with infections, autoimmune diseases, and immunodeficient states [2-5]. Moreover, there are several clinical and histological variants that have been reported in the literature, making early definitive diagnosis even more challenging. Recognized clinical variations of SS include bullous SS, cellulitis-like SS, necrotizing SS, and generalized pustular SS. Histological variants include crytococcoid, histiocytoid, eosinophilic, lymphocytic, and normolipemic xanthomized subtypes [2]. The exact pathogenesis of SS is not fully understood but it is believed to involve a combination of immune dysregulation, hypersensitivity immune reaction, cytokine induction, and genetic susceptibility (e.g., *MEFV* gene mutations, 3q chromosomal abnormalities, and human leukocyte antigen (HLA)-B54) [2,5,6].

SS typically presents as an abrupt onset of tender, erythematous rash, accompanied by fever, and elevated neutrophil counts. The rash morphology is characterized by tender, erythematous, and edematous papules, plaques, pustules, and/or nodules, usually beginning on the upper extremities or scalp and spreading in a craniocaudal fashion. Cases of lesions limiting to acral surfaces and male genitalia have been reported [2]. It can also involve extracutaneous sites. Neutrophilic infiltration of other organs can lead to extracutaneous manifestations such as ocular inflammation, arthralgias, colitis, hepatitis, alveolitis, diffuse alveolar hemorrhage, and in severe cases, meningitis and encephalitis. Ocular manifestations are common and can range from mild conjunctivitis to severe presentations such as bilateral panuveitis, central retinal artery occlusion, hemorrhagic conjunctivitis, meibomitis, and retinal detachment [2]. These extracutaneous manifestations can have significant morbidity and mortality, making early recognition and treatment crucial.

The diagnosis of classical or malignancy-associated SS requires meeting both major and two of four minor criteria developed by von den Driesch in 1994 [6,7]. Major criteria include (i) acute onset of tender erythematous plaques/nodules and (ii) histologic evidence of dense neutrophilic infiltrate without evidence of vasculitis. Minor criteria include (i) fever greater than 38^o^C, (ii) an association with malignancy, inflammatory disease, pregnancy, or preceding infection or vaccination, (iii) response to corticosteroids or potassium iodide, and (iv) at least three out of four elevated inflammatory markers (ESR > 20 mm/hour, elevated CRP, WBC > 8000 cells/uL, or > 70% neutrophils). Drug-induced SS requires all five criteria that were developed by Walker and Cohen in 1996 [8]. These include (i) acute onset of tender erythematous plaques/nodules, (ii) histologic evidence of dense neutrophilic infiltrate without evidence of vasculitis, (iii) fever greater than 38^o^C, (iv) temporal correlation between drug administration and clinical presentation, and (v) clinical improvement after withdrawing drug or with corticosteroids.

Corticosteroids are the primary mainstay of treatment for SS as most patients are highly responsive to steroids. Treatment is typically initiated with prednisone at 0.5-1 mg/kg/day. Symptoms generally improve within 48 hours and the rash resolves in one to two weeks. Steroid therapy should be tapered over four to six weeks. In mild to moderate cases, SS may resolve spontaneously or with treatment of the underlying condition or discontinuation of the offending drug. For refractory cases or when steroid-sparing therapy is preferred, alternative treatments such as indomethacin, colchicine, acitretin, and biologics may be considered, though steroids remain the first-line treatment due to limited evidence supporting the efficacy of other options [2,6].

Our case illustrates the diagnostic challenges of SS, requiring extensive evaluation and multidisciplinary collaboration. In this case, the initial differential of SS included infection, drug-induced skin reaction, and leukemia cutis. First, SS can resemble various bacterial, viral, fungal, and mycobacterial infections, making early biopsy essential [6]. Early skin biopsy is crucial to confirm the diagnosis and avoid exposure to antimicrobials. Second, the patient’s recent initiation of azacytidine therapy raised concern for a drug-induced skin reaction adding complexity to the diagnosis [9]. Third, the potential progression of the patient's MDS to AML was a concern, leading to the consideration of leukemia cutis. For instance, a retrospective analysis of cutaneous specimens from six patients diagnosed with malignancy-associated histiocytoid SS revealed potential cases of leukemia cutis upon fluorescence in situ hybridization (FISH) analysis [10]. This highlights the difficulty in distinguishing between these two entities. This is particularly relevant due to the similarity in presentation between leukemia cutis and SS, both of which can feature erythematous papules and nodules [11]. The biopsy eventually confirmed SS, but an earlier intervention could have expedited both diagnosis and treatment. It is noteworthy that SS associated with cancer tends to be more severe compared to cases in patients without malignancy [12]. Lastly, our case illustrates that SS, usually associated with elevated WBC, can also occur in the context of therapy-related neutropenia, such as that induced by azacytidine. Literature has documented similar cases of malignancy-related SS associated with chemotherapy-induced neutropenia [12,13]. This emphasizes the need to increase awareness of this atypical presentation.

## Conclusions

This case serves as an educational tool for hospitalists to enhance recognition of this rare pathology, which can easily be overlooked. Although the diagnostic criteria for SS are well-established, timely diagnosis and treatment remain challenging due to its broad differential diagnosis, especially with the increasing recognition of variants, associations, and overlapping features with other disease states. Given the rarity and complexity of SS, continued reporting of cases is crucial for improving recognition and diagnosis. For patients with known risk factors for SS, such as malignancy or new drug exposure, who present with symptoms of ocular complaints, arthralgias, or rash, including SS in the differential diagnosis is important, even if the patient is neutropenic. Early recognition can help prevent unnecessary advanced radiological imaging, reduce antibiotic exposure, and medical expenses, and ultimately improve patient outcomes by minimizing morbidity and mortality.
